# Notes of the Week

**Published:** 1921-05-07

**Authors:** 


### NOTES OF THE WEEK.

### THE PRINCE AMONG THE CHILDREN.

H.R.H. made his first appearance as President of the Hospital for Sick Children, Great Ormond Street, at the Court of Governors on Tuesday, May 3. Previously he paid a visit of inspection throughout the institution, where it will be remembered Princess Mary trained as a nurse for some two years. As Mr. John Murray, the Vice-Chairman, pointed out, such visits from the Prince are not only of great cheer, but a real and substantial help in the times of trouble and depression through which the hospital world is passing. In the case of the Hospital for Sick Chrildren there is a deficiency between ordinary income and expenditure of about £17,000, and the scheme to provide a country hospital still awaits funds before even a start can be made. Sir Arthur Lucas, who has been Chairman of the Committee for thirty-one years, is retiring, and as a tribute to his eminent services to the hospital he has been elected a Vice-President. An interesting, illustrated short history of the hospital has been published, which the Secretary will send on application.

### CHANGES AT ST. BARTHOLOMEW'S.

At a special meeting of the governors of the Alexandra Hospital for Children with Hip Disease, late of Queen Square, held last week it was resolved to enter into negotiation with St. Bartholomew's Hospital with a view to amalgamation. The Alexandra Hospital at present occupy the Swanley Convalescent Home buildings of St. Bartholomew's, which were used for military patients during the war. Presumably because of their considerable trust funds, the Governors of St. Bartholomew's have taken the step of obtaining an order from the Charity Commissioners respecting a scheme of payments by patients in which it is provided that not more than 100 beds in the "best wards" may be used for patients paying not more than £6 6s. a week, and for the other beds, after not less than 100 are set aside for the treatment of free patients, not more than £2 2s. a week may be charged. This order is for five years from January 1, 1921, but the new arrangement did not start until April 1. Assuming that the scheme will produce £25,000 a year, there will still be a deficiency, says the annual report, of about £45,000 a year. The Peace Year Appeal has been closed and the balance of £65,201 transferred to the Income and Expenditure Account, which also shows a gift of £10,000 from the National Relief Fund. The hospital now relies on a permanent appeal department, under the chairmanship of Sir Gordon Campbell, with as secretary Mr. Herbert Bloye, C.B.E., who had charge of the appeal work at the British Red Cross Headquarters during the war.

### DR. ADDISON AT THE FEDERATION'S DINNER.

Dr. Christopher Addison will be the chief guest at the annual dinner of the Federation of Medical and Allied Societies in recognition of the services he has rendered as first Minister of Health in circumstances of exceptional difficulty. The dinner, which takes place at the Café Royal on Thursday, May 26, will also be attended by the present Health Minister, Sir Alfred Mond, members of the Medical group in Parliament, and other notable guests. In view of the very great interest that is being taken, early application for tickets should be made to the General Secretary of the Federation at 5 Vere Street, London, W. 1.

### TUBERCULOSIS REGULATIONS, 1921.

Attention is to be drawn to these regulations, which came into force on May 1. For the corresponding paragraphs in the 1912 regulations substitutions have been made as required by the termination of sanatorium benefit on April 30. By the revised Article 3 the medical officer of a tuberculosis dispensary has access to the register of notifications mentioned in Article 4. This register used to be revised from time to time, but now it must be dealt with not less often than once every quarter, so that entries may be removed relating to persons who have died or ceased to reside within the district of the medical officer of health concerned. To facilitate this process it is urged in a circular letter that tuberculosis officers should, for the purpose of the regulations, act as officers of the sanitary authority under the direction of the medical officer of health. Article 5 requires that every medical officer of health shall notify movements and conditions of known tuberculosis subjects to his colleagues in those districts to which such persons move.

### THE DREADNOUGHT HOSPITAL.

At the recent dinner at the Hotel Cecil in celebration of the centenary of the above hospital Dr. Russell Wells, in proposing the toast of the evening, drew an apt comparison between 1921 and the year in which the hospital was founded. Then, as now, we had come through a great war—the battle of Waterloo. Then, as now, we had been dependent upon our Navy for many things. But one great difference marked the respective times: our forefathers had to pay the heavier debt in wounds, or in their consequences rather. In such circumstances in 1821 the Seamen's Hospital was founded solely with a view to the relief of great suffering. The magnitude of its achievement is shown by the fact that in the past century of its service over 200,000 seamen have passed through the hospital and over twice that number have been treated as out-patients.

### DR. DILL'S SCHEME IN LONDON.

The British Medical Association have circulated particulars of an insurance scheme for London on the lines of that originated in Brighton by Dr. Gordon Dill. In it the hospitals are proposed to be used as a consultative service by general practitioners, and in addition facilities are provided for dental treatment, nursing, massage, electrical treatment, and aids to diagnosis by means of laboratory and $x$-ray examinations. The scheme is so complete that visits of consultants are included. In this way the humble member who pays his £1 a year or more according to the size of his family is with them provided with aid in sickness equal to any that money can buy. It is estimated that the total income under the organisation might be £1,200,000, of which £585,000 would go to voluntary hospitals, and £195,000 to the staff fund of these institutions. The scheme does not interfere with the National Health Service or the general practitioner, as it does not provide members with ordinary medical attendance or other benefit to which they are entitled from the State or local authorities; it is worthy of the most careful consideration due to such practical and interesting constructive work.

### TOBACCO AND HEALTH.

It is refreshing to hear so candid and instructive a speech as that delivered last week by Sir James Cantlie on "Tobacco and Health." A short time ago we ventured to comment similarly upon the drinking of tea, urging the rational use of the beverage, but preserving also an appreciation of its failings. Similarly would Sir James have us consider smoking. His words have already been widely quoted, but the outstanding point is that smoking need not be injurious. It is not a habit to be absolutely and utterly condemned—if only the world smokes intelligently.

### REMARKABLE PROGRESS AT LEEDS.

In spite of much excellent work for hospitals, Leeds is not currently quoted, in company with Leicester or Newcastle, as a shining example of the value of a workmen's hospital fund. Yet the report for 1920 records that the fund raised nearly £40,000, and, what is more, has risen by nearly £14,000 over the total secured in 1919. The main source of income—namely, workshop collections—was chiefly responsible for this remarkable increase. Leeds General Infirmary received a grant of £20,000—a sum equal to the aim of the entire fund only three years ago! And the gross total would have been larger by at least £3,000 if unemployment had not occurred to diminish it. It is thus clear that though the primary object of the fund is to maintain three convalescent homes, it is of the greatest service to the Leeds hospitals. The General Infirmary is now spending over £90,000 a year, and is also overcrowded with a waiting-list of 700. The best hope of equalising this expenditure is Mr. Braime's scheme for employers' contributions, and the aim of this fund should be to produce a sum similar to that subscribed by the workmen. Over 650 firms are now on Mr. Braime's list, which will shortly include firms on the outskirts of the city.

### A "CRISIS PASSED" AT NOTTINGHAM.

Notwithstanding a deficit of £15,000, the Nottingham General Hospital has had a remarkable year. Donations, subscriptions, Hospital Saturday and Hospital Sunday showed healthy increases, which amounted to over £11,000 in all. Mr. F. Acton and Mr. Forman both declared that the crisis seems to be over in Nottingham, and that the voluntary system is safe there. A special tribute was paid to the help and goodwill of the miners. The appeals made last year were successful, and it is believed that the increases of income will be maintained, and that the same amount of effort can increase them. This is much to be hoped, for the hospital needs many things, including a pathological department and a modern nurses' home.

### HELP FROM THE UNEMPLOYED.

The Swansea General Hospital has latterly received a fine and growing response from the workers, whose contributions since 1914 have grown from £9,000 to £22,500. As always, the indirect effects of such widespread interest are many, one of the most remarkable of which appears to be the undertaking, lately mentioned by Mr. Roger Beck, whereby the workmen of Pontardawe and Neath have promised, "when they have returned to work," to make good what the hospital has lost through unemployment. The spirit thus shown is magnificent, for unemployment is apt to be demoralising and to limit all sensation to the need for pecuniary prudence. We hope that the "promise," to use Mr. Beck's word, will be made widely known, so that in Cardiff and other industrial centres this signal example of public spirit may be held up to admiration and to imitation.

### A MATRON'S FLAT.

Before long it is hoped to start rebuilding the Samaritan Hospital for Women, Upper Parliament Street, Liverpool. A sum of £12,000 is in hand, and most of the property on the site has been acquired. The plans have been drawn by Messrs. Edmund Kirby and Sons, architects, of Liverpool, and the building will face the end of the cathedral. The new hospital will contain an out-patient department in a lower ground floor, because of the hill, four general and five private wards, and the accommodation will include a "matron's flat"; the details of this will be studied with interest. The nurses, however, are to have their dining and sleeping rooms in the main building, a plan which only exceptional circumstances can justify in a new hospital. Even the laundry will be in the main building, which suggests a cramped site, and reminds us of the difficulty of building complete hospitals in the centre of great cities.

### THE HOSPITAL OF LAMBETH.

The plan to increase the number of beds in the Lambeth Guardians' Infirmary in Brook Street from 656 to 956 by using part of the adjoining workhouse is now being made permanent by a definite amalgamation. The enlarged institution will be called the Hospital of the Parish of Lambeth. The able-bodied inmates will be removed to the institution in Gordon Road, Camberwell. These facts seem to prove that the amalgamation is one of buildings only. It would be a definite set-back, of course, to amalgamate infirmary and workhouse. Indeed their separation alone has made progress possible.

